# Physical Activity and Exercise Participation among Malaysian Children (Able-Bodied vs. Physical Disability): A Cross-Sectional Study

**DOI:** 10.3390/children9050704

**Published:** 2022-05-11

**Authors:** Maziah Mat Rosly

**Affiliations:** Department of Physiology, Faculty of Medicine, Universiti Malaya, Kuala Lumpur 50603, Malaysia; maziahmr@um.edu.my

**Keywords:** questionnaire, disability, pediatric, quantitative research, rural health

## Abstract

Globally, physical activity levels (PAL) among able-bodied and children with a form of disability remain low. This study aims to characterize PAL and identify the demographic variables affecting children from partaking exercises to promote active lifestyles. Methods: The Physical Activity Scale for Individuals with Physical Disabilities questionnaire was used for the study. A total of 140 data responses were collected online or physically via passive snowball recruitment and quantitatively analyzed. Results: Five factors were extracted from the dimensions, consisting of household chores, household maintenance, high intensity exercise training, miscellaneous activities and school-related activities. Able-bodied children were significantly (*p* = 0.000) more active (median = 15.05, IQR = 13.06) than children with physical disabilities (median = 3.09, IQR = 2.58). The B40 household group reported significantly (*p* < 0.05, MET < 5.16/week) lower participation in health-beneficial (moderate-vigorous intensity) exercises as recommended by international guidelines. Conclusion: Children with physical disabilities reported significantly lower education achievements and PAL compared to their able-bodied counterpart. The majority of Malaysian children (69.3%) surveyed did not achieve the recommended aerobic exercise prescription.

## 1. Introduction

In children, which includes adolescents, defined as those below the age of 18 years old, active living and exercise participation are integral aspects of their development, fitness performance and overall well-being [1,2]. Various international guidelines have recommended that children and adolescents between the ages of 6 to 17 years old, perform approximately 60 min or more of moderate-to-vigorous physical activities (PA) daily [3,4,5]. However, global estimates have reported that only one in five children met these recommended PA lifestyles, with girls prone to being more sedentary than boys [6]. The increasing sedentary lifestyles among children and adolescents since the 21st century [6,7], raised concerns due to the higher risks of developing secondary, previously considered adult-onset diseases, such as obesity, diabetes mellitus and hyperlipidemia [8]. The disease complications can lead to lower productivity, higher morbidity, and faster mortality rates in children and adolescents [2,8].

In children with physical disabilities, opportunities to partake in PA that promote health-beneficial exercises, within moderate to vigorous intensities, are limited, as they often face common [9,10] and unique [11,12] barriers to participate in exercises for improved health parameters. The support of parents, teachers, therapists and health professionals become key influencers in changing the dynamics of this epidemiology [13]. Among the more commonly reported socio-environmental issues were parental support, access to facilities and lack of equipment or transportation [14,15]. These barriers contribute to lowered PA levels and puts them at risk for the development of secondary complications related to sedentarism [4,16].

Global and national guidelines on PA [16], are important components in a comprehensive and coherent governance, along with policy framework for public health action [4]. However, relevant data on specific targeted populations, in this case children and adolescents with physical disability, remain scarce, especially among developing and underdeveloped countries [7]. An assessment of the needs and unmet needs of individuals with physical disabilities are often overlooked [17,18], as specialized expertise trained to guide students with physical disabilities require both dedication and funding.

Currently, with the arrival of the COVID-19 pandemic, parents, teachers and therapists alike are facing difficulties ensuring adequate PA at home, where schools are rapidly shutting down globally [19,20]. This leads to longer durations of screen time among children and adolescents, altogether promoting higher incidences of sedentarism when people are mostly confined in their own home [19]. In addition, poorer families are greatly affected, since their marginalized social circumstances do not allow for active participation in exercise or sports due to limited amenities or equipment facilities at home [21,22]. Several other factors also have a lasting impact on a child’s PA participation, most importantly, their physical functional limitations, as well as family resources [23]. To date, very few studies have compared PA levels and exercise participation rates between able-bodied and children with disability [24], and even lesser studies had the epidemiological data on developing and underdeveloped countries. Considering health-related quality of life in children had been strongly associated with sports participation and active living [25], it is therefore imperative to conduct a cross-sectional demographic-based study on the PA status of children and adolescents in Malaysia.

The purpose of this cross-sectional study is aimed at looking into two objectives. The first is to assess and characterize overall PA levels and exercise participation rates among children in Malaysia, according to the global recommendations. The second objective is to compare and contrast factors that affect PA levels and exercise participation between different groups of children, taking into account their physical limitations, socio-demographic profiles or environmental factors within a cross-sectional survey. The initial hypotheses assume that PA levels and exercise participation rates are higher among able-bodied children compared to children with a physical disability. The findings of this survey, while noting any significant factors that may have contributed to PA and exercising behavior, may help in outlining frameworks to initiate an intervention plan that is designed specifically for children and adolescents in Malaysia.

## 2. Materials and Methods

### 2.1. Questionnaire and Pilot Survey

The demographic survey and Physical Activity for Individuals with Physical Disabilities (PASIPD) questionnaires were adapted from a previously validated questionnaire designed for both able-bodied and individuals with physical disabilities [26] within a Malaysian-based setting [27]. Minor revisions were made following the pilot survey [28], for items 12 and 13 of the PASIPD questionnaire to fit the target population of children and adolescents. For item 12, the description “taking care of another sibling” was added to fit a large family scenario in many Malaysian children’s households. In item 13, the “career work” description was substituted with schooling or learning activities in institutions, in order to fit the new targeted population. All revisions were made following the same protocols outlined in a previous study, which used language appropriate for children 12 years and above [27]. The pilot PASIPD survey was conducted among eight pre-test respondents, consisting of adolescents, professional therapists and a linguist, using a dichotomous scale of either a ‘clear’ or ‘unclear’ rating [29,30]. Feedbacks were collected and the questionnaire was revised back and forth for improvisation until an inter-rater agreement of more than 80% per item was achieved [29]. The revised questionnaires were then finalized for distribution following the pilot survey.

The PA levels using the PASIPD questionnaire is described in metabolic equivalents of tasks (MET) hours per day and requires respondents to recall their activities of daily living for the past seven days. It is an objective measure that captures the frequency (never, seldom: one to two d/week; sometimes: three to four d/week; or often: five to seven d/week) and duration (<1 h, 1 but <2 h, 2–4 h, >4 h) of activities conducted under 12 different categories, for able-bodied individuals and those with physical disabilities. A detailed description of the questionnaire can be found in Washburn et al.’s development and evaluation paper [26].

### 2.2. Participation Selection and Data Collection Procedure

The protocol for this ethics application was approved by the Universiti Malaya Research Ethics Committee (UMREC-995) on 11 September 2020. Children and adolescents aged between 7 and 17 years old, with no cognitive deficits affecting verbal or written command were eligible to participate. Children and adolescents within the physical disability category can include those with cerebral palsy, spina bifida, amputations, spinal cord injury or blindness, as suggested by Washburn et al. [26]. However, they must be able to understand the national language Bahasa Malaysia. Individuals with a form of cognitive or intellectual disability such as down’s syndrome and attention deficit hyperkinetic disorders were not eligible. Recruitment calls were carried out physically by passive “snowball recruitment” (refers to a non-probability sampling derived by recruiting future participants among acquaintances from existing participants through word of mouth) or online through various media links. All Malaysian children from various states in Malaysia were invited to participate. Informed written consent was taken from the parents or guardians prior to the start of the questionnaire.

The adapted questionnaires were also distributed physically and digitally to parents or guardians of potential respondents aged 12 years and below [31]. Respondents who require physical support while attempting the questions were assisted by a research assistant familiar with the questionnaires. To improve the accuracy of the responses given for the questionnaire, children aged less than 12 years old are supported by their parents or guardians during the data collection interview. It has been reported that children aged eight years or younger may have cognitive limitations in accurately responding to survey questions [32], and thus adult supervision and assistance are recommended.

### 2.3. Data Analysis

Data collected from the questionnaires achieved 140 completed responses. All data were analyzed using Excel 2019 (Microsoft Corporation, Redmond, Washington) and SPSS version 25 (IBM, Armonk, New York, NY, USA). Normality tests were conducted for the ordinal and continuous data collected using Shapiro-Wilk and Kolmogorov-Smirnov tests. PA levels in MET hours/day indicated non-normal distribution with *p* < 0.05 for both normality tests, while age indicated normal distribution in the histogram with *p* > 0.05.

Age and MET hours/day for total and individual PA were compared using independent T-test and Mann-Whitney U test between the able-bodied and physical disability groups, respectively. Some of the original demographic groups in Table 1, were coded into binomial categories for (a) age (children: 7–12 years old versus adolescents: 13–17 years old), (b) area (rural versus urban), (c) state (Klang Valley vs. others), (d) type of house (good versus poor accessibility i.e flats/wooden houses, terraces, others), (e) education level (non-formal versus formal), (f) household income (B40 versus M40 and T20), (g) type of mobility aid used (with or without aids), and (h) type of disability (cerebral palsy versus others). The income brackets were recategorized into B40 group (income ≤ 4999) and M40/T20 group (income ≥ 5000) in accordance with the Malaysian Department of Statistics recommendations [33].

Items in the PA scales were then analyzed for dimension reduction. Principal component dimension extraction was conducted separately based on able-bodied versus those with physical disability, as well as when combined. Factor analysis and determination were conducted using a univariate descriptive with varimax orthogonal rotations. The number of factors extracted was based on the criteria of an eigenvalue ≥1 and factor loading ≥0.4 [26,27,34,35]. However, factor analysis and extraction could not be computed for the physical disability group due to having one or less variables. The criteria for children and adolescents fulfilling the recommended PA guidelines were based on performing ≥5.16 MET hours/day of moderate-vigorous exercises. This is based on accumulation of items 4 (moderate exercise) and 5 (vigorous exercise) of the PASIPD questionnaire, having performed at least five to seven days (often) per week at 1 h to less than 2 h per day minimum. The significance of all tests was set at *p* < 0.05.

## 3. Results

### 3.1. Factor Component Dimension Extraction

Five factors were extracted from the dimension reduction analysis in both the combined and able-bodied groups. The PA dimensions extracted for able-bodied children (Table 2) were Factor 1: household chores (leisure activities, light and heavy housework); Factor 2: household maintenance (home repair, lawn and gardening work); Factor 3: high intensity exercise training (vigorous exercise and endurance training); Factor 4: miscellaneous activities (moderate exercise and caring for another person); and Factor 5: school activities (light exercises and school work).

The combined version of PA dimensions extracted (Table 3) consisted of Factor 1: household maintenance (home repair, lawn and gardening work); Factor 2: household chores (leisure activities, light and heavy housework); Factor 3: high intensity exercise training (vigorous exercise and endurance training); Factor 4: education-related activities (moderate exercise and school work) and Factor 5: miscellaneous activities (light exercise and caring for another person).

### 3.2. Combined Results Description

A total of 70 able-bodied children and 70 children with a form of physical disability were recruited. The demographic profiles of the respondents recruited were described in detail in Table 1. A comparison in Table 4 described the PA performed by both groups. Children (aged 7–12 years old) were less active than adolescents (13–17 years old) overall (*p* = 0.001), partaking in lesser PA associated with leisure (*p* = 0.41), vigorous exercises (*p* = 0.000), endurance training (*p* = 0.039), light (*p* = 0.001) and heavy (*p* = 0.007) houseworks, and school activities (*p* = 0.10). The able-bodied group (median = 15.05, IQR = 13.06) were more (*p* < 0.000) physically active compared to those with physical disabilities (median = 3.09, IQR = 2.58).

There was a significant difference seen between residents within the Klang Valley and other areas (*p* < 0.05). Respondents from areas outside the capital and satellites cities of Malaysia reported higher total MET hours/day (PA level, *p* = 0.009), light exercise (*p* = 0.024), moderate exercise (*p* = 0.005), light (*p* = 0.008) and heavy housework (*p* = 0.007), as well as lawn (*p* = 0.011) and gardening work (*p* = 0.023). Similarly, those coming from the B40 household group reported significantly lower activities in total MET hours/day (PA level, *p* = 0.001), light (*p* = 0.006), moderate (*p* = 0.035) and vigorous (*p* = 0.000) exercises, endurance training (*p* = 0.026), housework (*p* = 0.001), gardening (*p* = 0.002), and caring for another person (*p* = 0.003).

Finally, unsurprisingly, children dependent on a form of mobility aid were evidently significantly (*p* = 0.000) less active in all PA than their counterpart who did not require any walking aids. Interestingly, houses with better accessibility showed a significantly higher PA level in total MET hours/day, vigorous exercises (*p* = 0.034), endurance training (*p* = 0.003), and heavy housework activities (*p* = 0.049). Being in an urban or rural area, however, did not seem to affect PA levels in children or adolescents. Overall, only 69.3% of the 140 children and adolescents surveyed achieved the recommended PA levels.

### 3.3. Able-Bodied Children Results Description

Being in an urban or rural area, different states, or level of education did not significantly affect a child’s participation in any of the PA assessed. However, houses with good accessibility were found to significantly (*p* = 0.019) improve endurance training participation and frequency among able-bodied children. Additionally, being in the M40/T20 group were shown to significantly (*p* = 0.034) reduce moderate exercise participation and frequency, but not for other intensities.

A total of N = 43 (61.4%) of the 70 children and adolescents fulfilled the recommended PA guidelines in moderate-vigorous exercise participation per week. Fulfilling the recommended PA needed was not significantly (*p* > 0.05) associated with any known demographic factors such as sex, age, state, area, education level, type of house, or household income group.

### 3.4. Children with Physical Disability Results Description

The physical disability group had 58 respondents with cerebral palsy, 1 stroke, 1 amputee, 1 blindness, and the remaining 9 under other unknown etiology. Children with cerebral palsy reported significantly lower participation in moderate exercise (*p* = 0.007), light and heavy housework (*p* = 0.002) and gardening (*p* = 0.002).

Klang valley residents were less likely to participate in gardening activities (*p* = 0.035), but there was no significant difference in PA performed between urban and rural residents. Children within the B40 household income performed more light exercises (*p* = 0.001) but lesser gardening activities than the M40/T20 group. Interestingly, houses with poorer accessibility showed higher participation (*p* = 0.041) in light exercises as compared to those with better access.

Lastly, children with physical disability who are dependent on a form of mobility aid tend to perform fewer moderate exercises (*p* = 0.002), light (*p* = 0.000) and heavy (*p* = 0.000) housework and gardening (*p* = 0.000) activities. None of the 70 children and adolescents with physical disability fulfilled the recommended PA guidelines in moderate-vigorous exercise participation per week.

## 4. Discussion

The main significant finding from this cross-sectional study is the evidence of marked sedentarism seen in children with physical disability compared to their able-bodied counterpart in a Malaysian setting. Moderate-vigorous aerobic exercises, deemed important for fitness, health and overall well-being, were almost never performed by this sample population of children with physical disability. The findings from the study echoed similarly from surveys in other countries such as Australia [15,36], United States of America [37], Canada and Dutch populations [38]. In a small sample population of 34 children with disabilities in New South Wales, Australia, only 27% reported having fulfilled the amount of PA recommended. The studies highlight various serious health concerns within the population, as marked sedentarism is significantly linked to mental health illnesses such as depression, low self-esteem, poor cognition and low cardiorespiratory fitness [4,39]. This situation seems to also affect sports and exercise participation into adulthood, as the involvement rate among adults with physical disabilities in Malaysia still dwindles [28,40]. The negative attitude from the government, public and media regarding support and push for inclusivity were often cited as demotivators for this population [28,40]. In view of this, efforts must be made to encourage, support and motivate children with physical disability and those coming from the B40 household income group to partake in more health-beneficial exercises.

Another most unfortunate finding from this cross-sectional survey, is that all 70 children with a form of physical disability, reported having not completed or did not pass any formal compulsory education certification (deemed completing at least six years of primary school). It is possible that the relatively low household income brackets (B40) the children came from, makes it very difficult for them to gain access to good schools that support their special needs well. School support and expert teachers trained to handle the limitations presented by children with physical disabilities, often plague developed countries as well [41]. This report depicts the gravity of the situation in managing education in Malaysia, where children with disabilities are most often confined to their own home, with limited access to appropriate opportunities for formal education and social participation such as sports and exercise. The situation is further exacerbated by the arrival of the COVID-19 endemic, where physical schools are constantly shut down and migrating to online or hybrid learning. The procurement of necessary hardware or software to allow e-learning for the B40 group of students can be challenging and may further increase the intellectual gap compared to the M40/T20 groups.

The study found that partaking in moderate-vigorous aerobic exercise, within the recommended frequency and duration, for the able-bodied group, was not associated with any key demographical factors such as sex, age group, education level, type of house, or household income group. This may sound promising, as exercise participation rates can be improved with motivational training programs not limited to certain demographics. In this context, holistic efforts to shift the availability and feasibility of social PA, can be integrated into homes for health benefits. The World Health Organization’s International Classification of Functioning, Disability and Health model [23], provides a coherent view of how PA programs can be tailored according to contextual factors and bodily functions. With the arrival of the COVID-19 endemic, the closure of schools, training centers, and community-based facilities have limited physical-based participation in PA [21,42]. This leads to a paradigm shift, where PA promotions are converted into virtual, digital-based platforms that can reduce the gap between rural and urban residents [43,44].

Factor analysis of the PA reported, revealed that school activities were associated with moderate exercise in the able-bodied group and only light exercises in the combined group. This indicates that children with physical disabilities are less likely to participate in sports-related activities during physical education or do not partake in play activities that are intensive in nature. The findings from this study can explain a form of social isolation among children with physical disabilities, as they are not able to integrate well with other able-bodied children in schools during physical-based play activities. Poor sibling relationship, peer victimization, social stigmatism, isolation and bullying were known harmful experiences linked to children with disabilities [17,45]. Not being as physically capable as their peers and siblings may create rifts and physical gaps [46] that are exacerbated by various socio-environmental barriers [10].

Another important finding found in this study noted the low activity performed in household chores among children with physical disabilities. This indicated low independence among the children, which may lead to poorer self-confidence and personal growth later in adulthood. Low physical independence among children with physical disabilities have also been linked to malnutrition, poorer growth and lower school achievement [18]. Performing activities of daily living, such as household chores and maintenance, are important aspects to physical literacy. Physical literacy [47] constitutes aspects that are needed to value and take responsibility for engaging in PA through independent adulthood. This contributes to the fundamental development of movement competencies, confidence through the provision of positive challenges, and enhancing motivation for continued PA participation [48]. However, even able-bodied children coming from urban areas (especially within Klang Valley) were reported to not partake in household maintenance and chores, as reported in this study. This is most likely due to the common availability of domestic helpers/carers within high income households. In addition, a study by Wakely et al. [15] in Australia, reported children with a disability living in rural areas, often find difficulties in understanding their role within the society and family, with financial pressures adding to the parents burdening responsibilities.

Overall, only about 30.7% of the sample population surveyed fulfilled the recommended PA guidelines, and all of them were in the able-bodied group. The findings were much higher compared to a global epidemiological study (~70–84% did not achieve) [7], but lower among South East Asian regions (~10–20%) [6,7]. The 2030 *Global Action Plan on Physical Activity* [49] has adopted a new global target of reducing physical inactivity among children and adolescents by 15% come 2030. As mandated by the World Health Organization [1], each country should establish their own national guidelines in order to set PA targets achievable by the population. In the case of Malaysia, the prevalence of pronounced socio-environmental, political and economic barriers contributing to such sedentary lifestyles should be clearly understood prior to outlining intervention plans [28,40]. This is important as populations from developing countries face different and unique issues affecting their livelihood, progress and survivability [12,28]. For instance, in high income countries [50], children from higher socioeconomic status households were more likely to participate in sports and for longer duration, as opposed to the findings in this study.

In view of the profoundly low PA levels seen in children with physical disabilities in this sample population, urgent actions are needed to initiate appropriate measures to reduce their sedentary lifestyles. Therefore, research and interventions meant to improve exercise and sports participation for this marginalized population are warranted. Efforts can be initiated through the development of policies and guidelines [51] to implement appropriate training programs that can cater to their special needs. In order to do this, barriers and facilitators that can prevent and assist their participation in social activities should be researched [52], as a way to understand the issues that plagued them from leading active lifestyles. This notion goes in in line with the Sustainable Development Goals of 2030 [53], where infrastructure, amenities and transport are made safe, accessible and sustainable for all, with special attention to the needs of those in vulnerable situations, such as individuals with physicals disabilities.

## 5. Limitations

One major limitation of this study is the observational design, which provides only a one-time cross-sectional view of PA patterns of the children surveyed. This can present inaccurate findings that may have missed causal factors seen in interventional or cohort designs. However, the study has already taken into consideration the type of mobility aids used by the children with physical disabilities recruited in this study, which reported significant associations with low PA and non-participation to moderate-vigorous exercises. Additionally, the PA scale used for the study has reported relatively good test-retest reliability (ICC = 0.33–0.87), although the correlations to PA levels had been reported to be somewhere from poor to good (r = 0.22–0.51) [35,54,55]. Secondly, the findings of this survey were also limited by recall or reporting bias by respondents. This type of study bias is common among questionnaire surveys but is the more acceptable method of collecting demographic data on PA levels. Such errors in self-administered instruments can also include failure to read and/or understand instructions, as reported by Anderton et al. [56]. However, these errors can be reduced by adding interviewer-assisted format, to which the study has incorporated. Wearable devices such as accelerometers or pedometers lack harmonization across various types/brands and comparability to self-reported data [7,57]. As opposed to this, the PASIPD instead allows for description of PA levels in MET hours per day, which allows for absolute energy expenditure quantification that is systematic and has been standardized globally. Third, the study had limited findings from sample populations living in the Eastern part of Malaysia (Sabah and Sarawak states). The gravity of the situation, both in terms of education and PA levels among children and adolescents with physical disabilities, may be under-represented due to the lower earning potential reported for the two states [27].

Other limitations include technical specifications associated with the questionnaire selected for the study. The PASIPD questionnaire allowed for only an estimated amount of time performing aerobic moderate-vigorous exercises based on items 4 and 5 (moderate and vigorous aerobic exercises). The structure of the PASIPD questionnaire presents the hours spent on moderate-vigorous aerobic exercise per day in average MET hours [26,27], based on the compendium of physical activities tabulated [58,59]. This is opposed to the *Physical Activity Recall Assessment* [60] and *Leisure Time Physical Activity* [61] questionnaires, which reported the activities in minutes. In this study, the PASIPD questionnaire was selected in view of it having been validated for the Malaysian population, as well as designed to capture PA levels for both able-bodied individuals and those with physical disabilities [26]. This makes comparison surveys between different categories feasible in future work.

Finally, determination of any causal relationship between total PA level and moderate-vigorous exercise participation with the socioeconomic demographic factors associated could not be carried out since this was a cross-sectional study. Such causal correlations may assist in developing policies for schools that can be targeted for children with personal, domestic or socio-environmental barriers. Cohort designed future studies may be beneficial in determining effective intervention training programs for this population.

## 6. Conclusions

The cross-sectional survey comparing demographic profiles and activities of daily living between able-bodied and children with physical disabilities, depicts a situation where the latter is severely lacking in terms of education achievements, exercise participation, physical literacy and PA levels. The lack of inclusive opportunities to participate in health-beneficial exercises, daily activities promoting physical literacy and school integration between the two categories, indicated that those with physical disabilities were either neglected or disadvantaged. Overall, the majority of Malaysian children and adolescents (69.3%) surveyed did not achieve recommended PA levels set by the World Health Organization. Creative and innovative ways are needed to improve overall PA levels among children and adolescents.

## Figures and Tables

**Table 1:** Demographic profiles of respondents, N = 140.

Variable	Able-Bodied, (N = 70)	Disability, (N = 70)	Combined (N = 140)
N, sample size	70	70	140
Sex	Male: 39 Female: 31	Male: 43 Female: 27	Male: 82 Female: 58
Age, mean (SD)	12.5 (3.6)	12.9 (3.7)	12.7 (3.6) Male: 12.8 (3.4) Female: 12.7 (3.7)
Race	Malay: 70	Malay: 55 Chinese: 6 Indian: 9	Malay: 125 Chinese: 6 Indian: 9
State	Klang Valley: 17 Other states: 53	Klang Valley: 36 Other states: 34	Klang Valley: 53 Other states: 87
Area	Rural: 22 Small town: 27 Urban: 21	Rural: 25 Small town: 29 Urban: 16	Rural: 47 Small town: 56 Urban: 37
Type of house	Terrace: 33 Wooden House: 20 Flat: 3 Apartment/Condominium: 5 Semi-detached: 5 Bungalow: 4	Terrace: 26 Wooden House: 18 Flat: 6 Apartment/Condominium: 1 Semi-detached: 3 Bungalow: 2 Others: 14	Terrace: 59 Wooden House: 38 Flat: 9 Semi-detached: 8 Apartment/Condominium: 6 Bungalow: 6 Others: 14
Education	Did not complete formal education: 1 Primary school: 35 Secondary school: 34	Did not complete formal education:70	Did not complete formal education: 71 Primary school: 35 Secondary school: 34
Household income (in Malaysian Ringgit)	≤999: 1 1000–2499: 22 2500–3499: 14 3500–4999: 3 5000–9999: 20 ≥10,000: 10	≤999: 13 1000–2499: 27 2500–3499: 17 3500–4999: 5 5000–9999: 7 ≥10,000: 1	≤999: 14 1000–2499: 49 2500–3499: 31 3500–4999: 8 5000–9999: 27 ≥10,000: 11
Mobility aid	Nil	Manual wheelchair: 59 Crutches: 1 No aid: 10	Manual wheelchair: 59 Crutches: 1 No aid: 80
PAL (MET hours/day)	** 17.8 (12.1)	** 3.8 (3.2)	Male: 11.7 (12.4) Female: 9.6 (9.5)

** Significant difference at the level of *p* < 0.01. Abbreviations: MET, metabolic equivalent of a task; PAL, physical activity level. All values in mean (SD/IQR).

**Table 2:** Factor analysis of the Physical Activity Scale for Children (able bodied only).

Activity	Factor 1 (Household Chores)	Factor 2 (Household Maintenance)	Factor 3 (Exercise Training)	Factor 4 (Miscellaneous Activities)	Factor 5 (School Activities)
Leisure activities	0.54				
Light exercise					−0.62
Moderate exercise				0.78	
Vigorous exercise			0.81		
Endurance training			0.82		
Light housework	0.90				
Heavy housework	0.88				
Home repair		0.72			
Lawn work		0.76			
Gardening		0.81			
Caring for person				−0.69	
Work/School/Volunteer					0.76
Eigenvalue	1.98	1.97	1.55	1.41	1.18
Variance (%)	16.50	16.43	12.94	11.71	9.86
Cumulative variance (%)	16.50	32.93	45.86	57.58	67.43

**Table 3:** Factor analysis of the Physical Activity Scale for Children (combined).

Activity	Factor 1 (Household Maintenance)	Factor 2 (Household Chores)	Factor 3 (Exercise Training)	Factor 4 (Education -Related Activities)	Factor 5 (Miscellaneous Activities)
Leisure activities		0.60			
Light exercise					−0.66
Moderate exercise				0.78	
Vigorous exercise			0.77		
Endurance training			0.84		
Light housework		0.88			
Heavy housework		0.82			
Home repair	0.65				
Lawn work	0.80				
Gardening	0.86				
Caring for person					0.79
Work/School/Volunteer				0.70	
Eigenvalue	2.06	1.89	1.55	1.33	1.21
Variance (%)	17.13	15.72	12.91	11.10	10.10
Cumulative variance (%)	17.13	32.85	45.76	56.86	66.96

**Table 4:** Comparison of physical activities performed between children with and without physical disabilities.

Activities (in MET Hours/Day)	Able-Bodied, (N = 70)	Disability, (N = 70)	*p* Value	Combined (N = 140)
Leisure activities	2.7 (1.8)	2.0 (1.9)	** 0.019	2.3 (1.9)
Light exercise	0.9 (1.9)	1.0 (0.9)	0.000	1.0 (1.4)
Moderate exercise	2.9 (2.9)	0.1 (0.5)	0.000	1.5 (2.5)
Vigorous exercise	4.9 (6.6)	0.1 (0.8)	0.000	2.5 (5.3)
Endurance training	1.4 (3.9)	0.07 (0.5)	0.000	0.7 (2.8)
Light housework	0.73 (0.8)	0.1 (0.5)	0.000	0.4 (0.7)
Heavy housework	1.2 (1.6)	0.05 (0.4)	0.000	0.6 (1.3)
Home repair	0.5 (1.2)	0	0.000	0.3 (0.9)
Lawn work	0.5 (1.0)	0.09 (0.5)	0.000	0.3 (0.8)
Gardening	0.7 (1.7)	0.2 (0.7)	0.000	0.4 (1.3)
Caring for person	0.6 (1.1)	0.03 (0.2)	0.000	0.3 (0.9)
Work/School/Volunteer	0.81 (3.1)	0	0.004	0.4 (2.2)
Total	17.8 (12.1)	3.8 (3.2)	0.000	10.8 (11.3)

** Significant difference at the level of *p* < 0.05. Abbreviations: MET, metabolic equivalent of a task. All values in mean (IQR).
